# Review of *Online Dictionary of Invertebrate Zoology: Complete Work *by Mary Ann (Basinger) Maggenti, Armand R. Maggenti and Scott Lyell Gardner (editor)

**DOI:** 10.1186/1756-3305-1-38

**Published:** 2008-10-14

**Authors:** Les Chappell

**Affiliations:** 1 School of Biological Sciences, University of Aberdeen, Aberdeen, AB24 2TZ, UK

## Review

The revised fifth edition (version 2.1 posted July 2008) of this on-line dictionary of invertebrate zoology is available for free consultation on  (The Manter Laboratory and the University of Nebraska Digital Commons website). It is also possible to purchase the hard cover version  or the paperback .

The online version describes itself as 'the complete work' which gives access to all 963 pages on down-loading. It is also possible to down-load individual alphabetic entries, a facility that might be preferred by those with slow computing who may not wish to access the entire dictionary. The complete version is 963 pages long with approximately 12 entries per page. It is claimed to contain over 13,000 entries, and I am prepared to believe this. Where appropriate, each definition is accompanied by very brief notes on etymology and derivation as well as context of use; in addition, the authors state that they have used the original definitions wherever possible. The entries encompass the basic biology of 31 invertebrate phyla and this does seem to be a remarkably comprehensive reference work.

To make all entries readily accessible there is a page containing A-Z hotlinks to the appropriate section. This allows the browser to jump forward to the desired letter. Moving backwards and forwards through the text is achieved either by scrolling using the page arrows or by opening the bookmark facility on the left of the screen and selecting the letter of choice. Thus, navigating is straightforward once you are aware of these pdf reader buttons.

This is a very useful dictionary, one which I wish I had had access to when, as an undergraduate student of zoology, I was required to consult Hyman's intensely erudite prose and needed serious assistance in the explication of countless seemingly obscure terms. One obvious question for any new on-line database must be whether it reproduces other more generic databases. For instance, I selected rather at random a number of terms from the dictionary and entered them into a Google search. In each case I retrieved multiple responses as one might predict and, in some cases, I would have needed to have some prior knowledge to identify the most appropriate definition. Thus, although the internet now seems to be omniscient, a focused dictionary has considerable value. I therefore recommend that all invertebrate zoologists, old or young, mark the URL for this dictionary in their favourites section and use it whenever an unfamiliar (or even supposedly familiar) term is encountered or used.

I am of a vintage and disposition where I still prefer my reference books to be bound by card and printed on paper. Nevertheless, as I, like most of my colleagues, spend hours per day at my computer it seems obvious to use on-line resources in preference. I sigh at this a little since books feel and smell like friends whereas the World Wide Web has a slightly sinister side. It is, of course, quite feasible to print the on-line dictionary in your office and at four dictionary pages per printed page it is quite readable although obviously a little bulky at 240 pages of A4. Alternatively, you might wish to avail yourself of the printed version via the web-links as indicated above and at a price.

## Competing interests

The author declares that they have no competing interests.

